# Ovarian cancer survival population differences: a "high resolution study" comparing Philippine residents, and Filipino-Americans and Caucasians living in the US

**DOI:** 10.1186/1471-2407-9-340

**Published:** 2009-09-24

**Authors:** Maria Theresa M Redaniel, Adriano Laudico, Maria Rica Mirasol-Lumague, Adam Gondos, Gemma Leonora Uy, Jean Ann Toral, Doris Benavides, Hermann Brenner

**Affiliations:** 1 Division of Clinical Epidemiology and Aging Research, German Cancer Research Center, Heidelberg, Germany; 2 Manila Cancer Registry, Philippine Cancer Society, Manila, Philippines; 3 Department of Surgery, Philippine General Hospital, University of the Philippines-Manila, Manila, Philippines; 4 Department of Health-Rizal Cancer Registry, Rizal Medical Center, Pasig City, Philippines; 5 Department of Obstetrics and Gynecology, Philippine General Hospital, University of the Philippines-Manila, Manila, Philippines

## Abstract

**Background:**

In contrast to most other forms of cancer, data from some developing and developed countries show surprisingly similar survival rates for ovarian cancer. We aimed to compare ovarian cancer survival in Philippine residents, Filipino-Americans and Caucasians living in the US, using a high resolution approach, taking potential differences in prognostic factors into account.

**Methods:**

Using databases from the SEER 13 and from the Manila and Rizal Cancer Registries, age-adjusted five-year absolute and relative survival estimates were computed using the period analysis method and compared between Filipino-American ovarian cancer patients with cancer patients from the Philippines and Caucasians in the US. Cox proportional hazards modelling was used to determine factors affecting survival differences.

**Results:**

Despite more favorable distribution of age and cancer morphology and similar stage distribution, 5-year absolute and relative survival were lower in Philippine residents (Absolute survival, AS, 44%, Standard Error, SE, 2.9 and Relative survival, RS, 49.7%, SE, 3.7) than in Filipino-Americans (AS, 51.3%, SE, 3.1 and RS, 54.1%, SE, 3.4). After adjustment for these and additional covariates, strong excess risk of death for Philippine residents was found (Relative Risk, RR, 2.45, 95% confidence interval, 95% CI, 1.99-3.01). In contrast, no significant differences were found between Filipino-Americans and Caucasians living in the US.

**Conclusion:**

Multivariate analyses disclosed strong survival disadvantages of Philippine residents compared to Filipino-American patients, for which differences in access to health care might have played an important role. Survival is no worse among Filipino-Americans than among Caucasians living in the US.

## Background

Ovarian cancer is the second most common gynaecological cancer worldwide and the sixth most common cancer in women overall [1,2]. The majority of cancer cases occur in developed countries, and age standardized incidence and mortality rates are about two-fold higher in more affluent nations (10.2 and 5.7 per 100,000 population) as compared to less developed nations (5 and 2.9 per 100,000) [2]. However, there is large variation within both groups of countries. Within countries, ovarian cancer incidence and mortality have likewise been reported to vary between racial groups. The incidence rate of Philippine residents in 2002 was estimated at 11.5 per 100,000 [3] as compared to 10.3 for Caucasians and 8.9 for Asian and Pacific Islanders (API) in the United States [4]. Mortality rates were reported as 6.3 [3], 6 and 3.3 [4] per 100,000 for Philippine residents, Caucasians and APIs, respectively.

Comparisons in ovarian cancer survival between developed and developing nations, as well as between ethnic groups within countries, are few [5-7], but are important in determining sources of population survival discrepancies. In contrast to most other cancers, limited data from some developed and developing countries suggest that five-year relative survival rates were surprisingly similar, ranging from 31 to 42% and from 16 to 51%, respectively [2]. In the US, non-Hispanic white women were reported to have reduced risk of death as compared to African-Americans but have an increased risk compared to Filipino-American women [5]. However, previous comparative studies between developing and developed countries did not take into account potential differences in major prognostic factors, such as stage at diagnosis or morphology, which have been reported to vary between ethnic groups [5].

In this paper, we take a "high resolution" approach [8-10] to elucidate the role of factors not routinely available in population-based cancer registries, including ethnicity, stage at diagnosis, morphology, and access to treatment, in comparing ovarian cancer survival between Philippine resident patients, Filipino-Americans and Caucasians living in the US.

## Methods

### Databases

#### United States SEER 13

Using the Surveillance, Epidemiology and End Results (SEER) 13 database [4], ovarian cancer patients of Filipino-American or of Caucasian origin, including those of Hispanic ethnicity, were identified. Patients aged 15 and older, diagnosed with malignant ovarian cancer between January 1, 1993 and December 31, 2002 and followed with respect to vital status until December 31, 2002 were included in the study.

#### Manila and Rizal Cancer Registries

Patient information for residents of the National Capital Region (NCR) of the Philippines was abstracted from the Philippine Cancer Society-Manila Cancer Registry (PCS-MCR) and the Department of Health-Rizal Cancer Registry (DOH-RCR). The registries are regarded as among the high-quality registries from developing countries and have consistently been included in the "Cancer Incidence in Five Continents" series [11-15]. They follow cancer registration definitions and data collection guidelines set by the International Agency for Research on Cancer (IARC) and the International Association of Cancer Registries (IACR) [16].

Using the same inclusion and exclusion criteria as for the SEER databases, a list of 2,898 ovarian cancer cases diagnosed between 1998 and 2002 was generated, from which sub samples of 200 cases diagnosed in each calendar year of interest were randomly drawn using the .sample command in STATA version 6 [17]. Patients were followed with respect to vital status until December 31, 2002 as follows: survival status was assessed from death certificate notifications mentioning cancer as the cause of death, which were collected from Local Civil Registry Offices. For those not identified as dead, active follow-up by personal visits to the patients' last known place of residence were done to confirm vital status.

The project proposal was approved by the Ethics Review Board of the National Institutes of Health of the University of the Philippines Manila. The information obtained strictly conformed to the code of conduct stipulated by the Guidelines on Confidentiality for Population-based Cancer Registries [16].

### Data analysis

#### Estimation of Survival using Period Analysis

**Figure 1:**
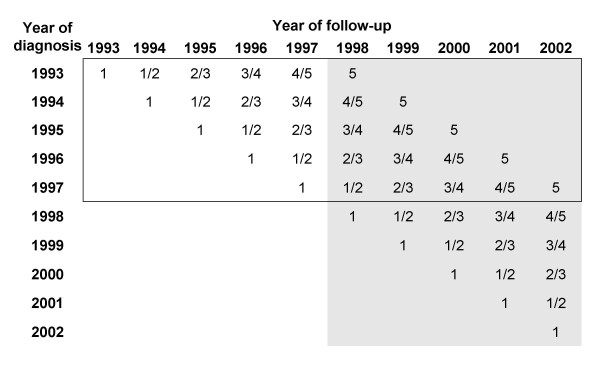
**Principle of period analysis**. Database used to derive the cohort analysis estimates (solid frame) and period analysis estimates (shaded area). The numbers within the cells indicate the years of diagnosis

To derive survival estimates, cohort-based analyses such as the conventional life-table (actuarial) method or the Kaplan-Meier method [18,19] have traditionally been used. With the cancer database including cancer incidence and follow-up data from 1993 to 2002, a cohort based analysis would typically have pertained to patients diagnosed in 1993-1997 and followed through 2002 (see figure 1, framed area). To derive more up-to-date survival estimates, we employed period analysis, a new method of survival analysis introduced by Brenner and Gefeller in 1996 [20]. Here, only the survival experience of patients for the 1998-2002 period was included (see figure 1, shaded area). This was done by left truncation of observations at the beginning of the period and right censoring at its end (i.e. at December 31, 2002 or the last date known alive). It has been consistently shown by multiple empirical evaluations that period analysis provides more up-to-date estimates of survival expectations for patients diagnosed in the respective period [21-25].

#### Estimation of Relative Survival

As commonly practiced in population-based cancer survival analysis, both absolute and relative survival rates were calculated. The relative survival probabilities are estimated as ratios of the observed survival of cancer patients and the expected survival of a group of people with the same age and sex distribution from the general population, and reflect the survival experience of cancer patients in the absence of competing causes of death [26,27]. Using the so-called Ederer II method [28], expected survival was derived from life tables for the year 2000. For the SEER populations, the life table for whites from the US National Center for Health Statistics [29] were used. The life table for the Philippine resident population was derived from the projected population estimate and the actual mortality data for this area, which were obtained from the Philippine National Statistics Office.

#### Age adjustment

To enable comparison of ovarian cancer survival estimates between different cancer populations, age adjustment was done. Age-specific period survival estimates (absolute and relative), using age groups 15-39, 40-49, 50-59, 60-69 and 70 and above, were first obtained from the Philippine resident, the Filipino-American and the Caucasian populations. The age-specific estimates were then weighted and summed for each population group, using weights from the World Standard Cancer Patient Population (WSCPP) [30].

#### Tests for survival differences between cancer patient populations

The differences between survival estimates for the three cancer patient populations were tested for statistical significance using a novel modelling approach for period analysis [31]. First, age-specific numbers of patients at risk and of deaths by year of follow-up were calculated for each population group. Poisson regression models were then fitted, wherein the numbers of deaths were modelled as a function of the population group (entered as a categorical variable), year of follow-up (1, 2, 3, 4, 5 - entered as a categorical variable) and age-group (as described above - entered as a categorical variable), using the logarithm of the person-years at risk as offset, and accounting for late entries and withdrawals as half persons, as described in detail elsewhere. This allowed for testing of significance of differences in survival, after adjustment for age and based on p-values for the population parameter estimate. A significance level of alpha = 0.05 (two-sided testing) was used.

#### Multivariate analysis

To explain possible survival differences and identify factors affecting survival, both within and between the three cancer patient populations, the Cox Proportional Hazards model was used. For each population group, bivariate associations of age, stage at diagnosis, histology, and receipt of surgery and of radiotherapy with survival were determined using individual Cox models. A multivariate model was then built jointly for all three groups to compare survival probabilities between populations. Relative hazards were calculated using Filipino-Americans as the reference group, while controlling for the effects of age, stage, histology, surgery and radiotherapy, first individually and then simultaneously. Those with missing information were excluded in the multivariate analysis. The assumption of proportional hazards for Cox models was checked by plotting the log of the negative log of the survival density functions vs the log of survival time. The plotted lines were roughly parallel over time and no violations of the proportional hazards assumption were found.

Age at diagnosis was categorized into the age adjustment groupings mentioned earlier. Stage categories were based on the Federation of Gynecology and Obstetrics (FIGO) stages I, II III and IV [32]. Histology was classified based on the International Classification of Diseases for Oncology (ICD-O) and the Systematized Nomenclature of Medicine [33] (serous, clear cell, endometrioid, mucinous and other types, including Brenner, granulosa cell, germ cell and sex cord stromal cell tumors). For the receipt of surgical treatment and of radiotherapy, binary variables (with/without) were used. A sub-analysis for the receipt of chemotherapy was done for all the Philippine residents included in the study, but chemotherapy and hormone therapy were not included in the Cox models as these were not available from the SEER public use database.

All analyses were done with the SAS Statistical Analysis Software. Special macros were used for standard and modelled period survival analysis as previously described [31,34].

## Results

A total of 463 Filipino-American and 22,290 Caucasian ovarian cancer patients were included in the analysis, after exclusion of around 1% of cases who are coded in situ and those who were identified by death certificates only (DCO). From the 2,000 randomly sampled patients from the Philippine databases, 1,475 ovarian cancer patients (73.8%) were included after 220 (11%) and 305 (15.2%) patients were removed due to invalid data, such as incorrect age, sex and primary site, and due to the absence of any survival time information, respectively. Of the included patients not known to be dead, complete 5-year follow-up information was obtained for 33.8%, while at least some follow-up could be ascertained for another 41.3%.

The distribution of cases by age groups, stage at diagnosis, histology, surgery and radiotherapy are shown for each population in Table 1. Caucasian patients were older than other groups, with more than 50% aged 60 or above while Philippine residents were youngest with more than 50% below 50 years. Around half of patients were diagnosed in the advanced stages (stages III and IV) in the Philippine resident population and Filipino-Americans, while the majority (66%) of Caucasians presented with advanced stage disease. Histologic profile varied, with Caucasians having the highest proportion of serous cancers (42%) and fewest proportions of endometrioid (14%) and mucinous (7%) types. Filipinos from the Philippines have the highest proportions of endometrioid (22%) and mucinous (24%) types with the smallest proportion of serous cancers (20%). Between 78 and 90% of patients underwent surgery and less than 10% received radiotherapy in all groups. Of the Philippine resident patients with known chemotherapy status, 24% received this treatment.

**Table 1:** Tumor characteristics of ovarian cancer patients, Philippine resident population, and Filipino-Americans and Caucasians from US SEER, 1993-2002


	**Philippine resident population**		**Filipino-Americans**		**Caucasians**		
**Variable**	**(N = 1475)**		**(N = 463)**		**(N = 22290)**		**p-value**
		
	**Freq**	**%^1^**	**Freq**	**%^1^**	**Freq**	**%^1^**	

**Age group**							
< 40	415	28.1	64	13.8	1545	6.9	< 0.0001
40-49	364	24.7	110	23.8	2948	13.2	
50-59	355	24.1	130	28.1	4356	19.5	
60-69	213	14.4	81	17.5	4787	21.5	
70+	128	8.7	78	16.9	8654	38.8	

**FIGO**							
I	316	33.5	160	35.9	4989	24.0	< 0.0001
II	112	11.9	62	13.9	2013	9.7	
III	315	33.4	138	30.9	8222	39.5	
IV	201	21.3	86	19.3	5609	26.9	
Unknown	531		17		1457		

**Morphology**							
Serous	236	20.1	130	30.2	8520	42.4	< 0.0001
Clear cell	72	6.1	44	10.2	974	4.9	
Endometrioid	259	22.1	83	19.3	2897	14.4	
Mucinous	285	24.3	45	10.5	1453	7.2	
Others	320	27.3	128	29.8	6255	31.1	
NOS^2^	303		33		2191		

**Surgery**							
With surgery	1214	88.6	396	85.7	17490	78.7	< 0.0001
Without surgery	157	11.5	66	14.3	4743	21.3	
Unknown	104		1		57		

**Radiotherapy**							
With radiotherapy	95	7.7	12	2.6	437	2.0	< 0.0001
Without radiotherapy	1144	92.3	445	97.4	21732	98.0	
Unknown	236		6		121		

^1^percentages refer to those with known categories

^2^includes: Neoplasms; Carcinoma, NOS; Carcinoma, Undifferentiated, NOS

Age adjusted and age-specific estimates of absolute and relative 5-year survival are shown in table 2. Five-year over all relative survival was within a narrow range (49.3-54.1%) in the three cancer patient populations. With few exceptions (which, given the relatively large standard errors in some of the age specific survival estimates, might be due to chance variation) relative survival decreased with age in all three cancer patient populations. Strong, statistically significant disadvantages in 5-year absolute and relative survival were seen in patients from the Philippines compared to Filipino-American patients in age groups 50-59 and 60-69.

**Table 2:** Five-year absolute and relative survival (in %) of ovarian cancer patients adjusted to the World Standard Cancer Patient Population, Philippine resident population, and Filipino-Americans and Whites from US SEER, 1998-2002


**Variable**	**(1) Philippine resident population**		**Between (1) and (2)**		**(2) Filipino-Americans**		**Between (3) and (2)**		**(3) Caucasians**	
					
	**%**	**SE**	**Diff**	**p-value**	**%**	**SE**	**Diff**	**p-value**	**%**	**SE**

**Absolute Survival**										
***Over all survival***	44.0	2.9	7.4	0.02	51.3	3.1	-4.6	0.29	46.7	0.5
***Age group***										
< 40	64.1	4.6	11.6	0.27	75.7	7.5	0.8	0.67	76.5	1.6
40-49	55.8	4.9	-1.1	0.99	54.8	6.7	7.9	0.11	62.6	1.3
50-59	38.2	4.8	25.8	< 0.001	64.0	5.9	-12.8	0.04	51.2	1.1
60-69	36.1	6.3	17.2	0.02	53.3	8.5	-13.4	0.13	40.0	1.0
70+	34.6	8.0	-12.7	0.03	21.9	6.0	-2.6	0.86	19.3	0.6

**Relative Survival**										
***Over all survival***	49.7	3.7	4.5	0.01	54.1	3.4	-4.8	0.33	49.3	0.5
***Age group***										
< 40	64.6	4.6	11.5	0.30	76.0	7.6	0.8	0.67	76.9	1.6
40-49	57.0	5.1	-1.6	0.99	55.4	6.8	8.0	0.11	63.4	1.3
50-59	40.0	5.0	25.8	< 0.001	65.8	6.1	-13.1	0.05	52.7	1.1
60-69	40.4	7.1	16.7	0.05	57.0	9.1	-14.1	0.14	43.0	1.1
70+	50.8	11.7	-23.0	0.03	27.8	7.6	-2.6	0.74	25.2	0.8

Diff, difference; SE, standard error

As shown in table 3, late stage at diagnosis and not receiving surgery were all strongly related to the risk of dying in each of the three populations. Furthermore, compared to patients with serous cancers, prognosis tended to be substantially better in patients with endometrioid, clear cell and mucinous cancer, and less favorable in patients with other forms of cancer. A sub-analysis among the Philippine residents showed that not receiving chemotherapy was also related to the risk of death (RR, 1.54; 95% CI 1.22-1.94).

**Table 3:** Relative risk of death according to various prognostic factors among ovarian cancer patients, Philippine resident population and from Filipino-Americans and Caucasians from US SEER, 1993-2002, Bivariate analysis


**Variable**	**Philippine resident population**		**Filipino-Americans**		**Caucasians**	
			
	**RR**	**95% CI**	**RR**	**95% CI**	**RR**	**95% CI**

**Age group**						
< 40	1.00		1.00		1.00	
40-49	1.26	0.94 - 1.68	1.82	1.01 - 3.28	1.78	1.57 - 2.02
50-59	1.80	1.37 - 2.36	1.31	0.72 - 2.36	2.50	2.22 - 2.81
60-69	2.06	1.52 - 2.78	1.84	0.98 - 3.44	3.54	3.15 - 3.97
70+	2.67	1.91 - 3.73	4.08	2.29 - 7.28	6.94	6.20 - 7.76

**FIGO Stage**						
I	1.00		1.00		1.00	
II	2.37	1.42 - 3.97	2.28	1.20 - 4.34	2.75	2.50 - 3.02
III	7.06	4.78 - 10.43	5.23	3.19 - 8.57	5.18	4.82 - 5.56
IV	14.28	9.59 - 21.26	12.54	7.60 - 20.70	9.08	8.45 - 9.75

**Morphology**						
Serous	1.00		1.00		1.00	
Endometrioid	0.80	0.48 - 1.31	0.51	0.27 - 0.98	0.61	0.54 - 0.68
Clear cell	0.85	0.61 - 1.18	0.50	0.30 - 0.83	0.51	0.48 - 0.55
Mucinous	0.67	0.47 - 0.95	0.46	0.23 - 0.90	0.61	0.56 - 0.67
Others	1.55	1.16 - 2.07	1.14	0.79 - 1.65	1.55	1.49 - 1.62

**Surgery**						
With surgery	1.00		1.00		1.00	
Without surgery	3.10	2.44 - 3.94	6.30	4.50 - 8.82	5.18	4.97 - 5.39

**Radiotherapy**						
With radiotherapy	1.00		1.00		1.00	
Without radiotherapy	1.25	0.87 - 1.79	1.20	0.44 - 3.26	1.25	1.09 - 1.43

RR, Relative Risk; 95% CI, 95% Confidence interval

In bivariate comparative survival analysis between population groups (table 4), substantial excess risk of death was seen among ovarian cancer patients from the Philippines and Caucasians as compared to Filipino-American patients. For Philippine residents, excess mortality was further increased when controlling for age and stage at diagnosis, and quite substantial excess mortality was found (RR, 2.45; 95% CI, 1.99-3.01) after controlling for these and other factors in multivariate analysis. The excess risk of Caucasian ovarian cancer patients compared to Filipino-American ovarian cancer patients was mostly explained by the age and stage differences, and no significant difference persisted in the multivariate analysis (RR, 1.17, 95% CI, 0.99-1.37).

**Table 4:** Relative risk of death for ovarian cancer patients from the Philippine resident population and for Caucasian patients compared to Filipino-American patients from US SEER, 1993-2002


**Variable**	**Philippine resident population**		**Filipino-Americans (reference group)**		**Caucasians**	
			
	**RR**	**95% CI**	**RR**	**95% CI**	**RR**	**95% CI**

**Bivariate analysis**	1.46	1.23 - 1.74	1.00	---	1.53	1.32 - 1.77

**After controlling for other variables**						
Age	1.82	1.53 - 2.16	1.00	---	1.17	1.01 - 1.36
FIGO Stage	1.77	1.48 - 2.13	1.00	---	1.19	1.02 - 1.39
Morphology	1.66	1.38 - 2.01	1.00	---	1.49	1.27 - 1.75
Surgery	1.44	1.21 - 1.71	1.00	---	1.41	1.22 - 1.64
Radiotherapy	1.60	1.34 - 1.91	1.00	---	1.55	1.34 - 1.80
**Multivariate analysis**^1^	2.45	1.99 - 3.01	1.00	---	1.17	0.99 - 1.37

RR, Relative Risk; 95% CI, 95% Confidence interval

^1^Controlling for all variables listed in the table

## Discussion

In this "high resolution" study comparing ovarian cancer survival in the Philippine resident and Filipino-American patients, sharing the same ethnicity, and in Filipino-American and Caucasian patients in the US, sharing the same health care system, overall survival differences were found to be relatively small. However, Philippine resident patients showed a more favorable distribution with respect to major prognostic factors, such as age, stage and morphology, and major excess mortality in this patient group was disclosed after control for these factors in age specific and multivariate analysis. By contrast, an apparent survival disadvantage of Caucasian patients compared to Filipino-American patients essentially disappeared after controlling for these factors. Taken together, these results point to the relevance of health care related factors for explaining survival differences.

Disadvantages in absolute and relative survival of Philippine residents compared to Filipino-Americans were particularly large for age groups 50-59 and 60-69, which can be explained to some extent by more unfavorable stage distributions. The proportions of patients presenting with advanced stages in age groups 50-59 and 60-69 were much lower in Filipino-Americans (48.4 and 55%, respectively) than among Philippine residents (60.4 and 69.2%, respectively).

In the absence of effective screening methods, only a small proportion of ovarian cancer cases are diagnosed in early stages, where surgery alone will be effective [32,35,36]. Most cases were diagnosed as advanced disease, where chemotherapy is administered either as neo-adjuvant or adjuvant treatment [32,35,36]. The proportion of those given chemotherapy among Philippines residents is much lower than an estimated 65% for US patients [32], which is likely to be reflective of both the larger proportion of cases in later stages in US patients, and the poor access to chemotherapy among Philippine residents.

In developed countries, survival rates have improved in the recent decades [37-40], mainly due to progress in treatment, including the development of adequate debulking surgical procedures and effective chemotherapy regimens. Even with advanced stage, patients with no gross residual after the surgical debulking have a considerably better prognosis than those with minimal or extensive residual. The number of residual sites also appears to be important [41]. Current chemotherapy protocols are based on the experience of developed countries and its effectiveness do not necessarily translate in developing country settings, given the differences in resources and attitudes [42]. The availability and affordability of drugs differ between the developed and developing world, and complex chemotherapy regimens might not be feasible or affordable in low income nations [42]. Furthermore, patients might not complete prescribed regimens due to financial constraints [42], as most chemotherapy drugs are paid for privately in developing countries [43].

In the NCR, Philippines, adjuvant chemotherapy has been shown to improve survival [44]. However, while chemotherapy is available and at par with the western world, the distribution of specialized centers offering cancer care services is not proportionate, with most located in the major cities [45]. Moreover, most state of the art diagnostic and treatment facilities are situated in tertiary private hospitals, which typically are expensive and beyond the means of average citizens. While subsidized services are provided by government institutes, these are limited and represent only a fraction of the total number of hospitals.

Compounding the problem among Philippine residents is the persistent disbelief in chances to be cured from cancer [46]. In spite of health information campaigns, many in the country still perceive cancer as a highly fatal disease that leads to eventual death. When faced with high costs, difficult and long treatment process, and low or unsure chances of survival, many patients opt to either refuse or discontinue any form of therapy when it becomes an excessive burden.

While much of the differences are likely to be explained by health care access, dissimilar distributions by age, stage and histology suggest a possible role of biological factors. The differences in tumor biology between populations might be reflective of the heterogeneous nature of ovarian cancer, and should be investigated in more detail, given that previous studies have shown significant variation of survival by histologic subtypes [5,47-49].

Between Filipino-Americans and Caucasians, slightly higher absolute and relative survival rates were observed for the former group. The distributions by age and stage at diagnosis vary between the groups, with Filipino-Americans having more favorable characteristics. The Cox model showed that the higher proportion of Caucasian women with older age and advanced disease explains most of the apparent survival difference. After adjustment for these variables, as well as morphology and treatment, the residual excess risk was small (15%) and not significant. Sensitivity analyses regarding difference in survival estimates between Caucasian population with and without Hispanics did not show a significant difference.

Our study is limited to variables that were available and comparable in the databases, and not all possible factors that could affect survival were considered. Information on chemotherapy is not included in the SEER public use database whereas some tumor characteristics such as grade, size and heterogeneity were not available from the Philippine database. In addition, socio-economic status, family history of ovarian cancer and lifestyle factors, like contraceptive use and hormone intake, were also not studied. Similarly, more detailed information on health care access, particularly on access to and availability of various treatment regimens, as well as application of treatment guidelines, protocols used, specialization and expertise of treating physicians, were not obtainable.

Patients with invalid data and those who do not have follow-up time were excluded from the study, but it is unlikely that they have higher survival than those who were included. Most such patients have incomplete records and were not traced to other hospitals in the NCR, indicating that they might not have consulted physicians after the initial diagnosis, or have had limited consultations afterwards. They most probably have not received any form of treatment as the availability of cancer treatment is limited outside the NCR. The survival rates presented might therefore overestimate true survival of Philippine residents to some extent.

## Conclusion

In conclusion, the results of the multivariate analyses disclosed strong survival disadvantages of Philippine residents compared to Filipino-American patients, despite similar overall survival rates observed in the presence of more favorable distributions of major prognostic factors. The survival disadvantage of Philippine resident patients most likely reflects differences in access to and affordability of effective health care and treatment, such as chemotherapy. Emphasis should therefore be given on improving access to and affordability of effective treatment regimens. Prognosis is no worse among Filipino-Americans than among Caucasians living in the US.

## List of Abbreviations used

API: Asian and Pacific Islanders; AS: Absolute survival; DCO: Death certificates only; DOH-RCR: Department of Health-Rizal Cancer Registry; EUROCARE: European **ca**ncer registry-based study on survival and care of cancer patients; FIGO: Federation of Gynecology and Obstetrics; RR: Relative risk; IACR: International Association of Cancer Registries; IARC: International Agency for Research on Cancer; ICD-O: International Classification of Diseases for Oncology; NCR: National Capital Region; PCS-MCR: Philippine Cancer Society-Manila Cancer Registry; RS: Relative survival; SE: Standard error; SEER: Surveillance, Epidemiology and End Results; WSCPP: World Standard Cancer Patient Population; US: United States

## Competing interests

The authors declare that they have no competing interests.

## Authors' contributions

The contributions of the authors are as follows: MTR contributed in the planning of the study, supervised data collection, performed the analysis and wrote the manuscript; AL, MRL, GU, JT and DB planned and supervised data collection, reviewed and edited registry abstracts, and performed data management; AG assisted in the analysis; HB contributed in the planning of the study and supervised data analysis and writing of the manuscript. All authors have read and approved the final manuscript.

## Pre-publication history

The pre-publication history for this paper can be accessed here:

http://www.biomedcentral.com/1471-2407/9/340/prepub
